# Efficacy of Memantine, Donepezil, or Their Association in Moderate-Severe Alzheimer's Disease: A Review of Clinical Trials

**DOI:** 10.1155/2013/925702

**Published:** 2013-10-29

**Authors:** Ivana Molino, Luisa Colucci, Angiola M. Fasanaro, Enea Traini, Francesco Amenta

**Affiliations:** ^1^Centro Ricerche Cliniche, Telemedicina e Telefarmacia, Università di Camerino, Via Madonna delle Carceri 9, 62032 Camerino, Italy; ^2^Unità Valutativa Alzheimer e Malattie Involutive Cerebrali, Azienda Ospedaliera Rilievo Nazionale A. Cardarelli, 80131 Napoli, Italy

## Abstract

*Background*. Acetylcholinesterase (AChE)/cholinesterase (ChE) inhibitors (Is) and memantine are licensed for symptomatic treatment of mild-moderate and moderate-severe forms of Alzheimer's disease (AD), respectively. High doses of the AChE-I donepezil were licensed in the USA for moderate-severe AD, and the association AChE/ChE-Is plus memantine was proposed for AD at this stage. *Objectives*. This paper has reviewed evidence from clinical trials of the effectiveness of memantine, donepezil, or the two drugs in association in managing moderate-severe AD. *Method*. Double-blind, placebo-controlled randomized trials (RCTs) using memantine or donepezil alone or in association versus placebo in moderate-severe AD were reviewed. Analysis done in January 2013 considered the years 2007–2012. *Results and Conclusion*. Only 83 of the 941 papers selected were considered relevant, and only 13 met the criterion of “adequacy and representativeness.” Memantine and donepezil lead to improvements in moderate-to-severe AD and the choice between the compounds should be based on their contraindications more than on disease severity. No evidence was found of advantages of the association of memantine-donepezil. The heterogeneity of conditions explored by RCTs, the relatively short time of observation (24–52 weeks), and the different cognitive assessment tools used did not allow comparing properly different trials.

## 1. Introduction

Alzheimer's disease (AD), the most common adult-onset dementia, is associated with very high costs for families and the society, as these patients need support and often institutionalization in the advanced stage [1]. Treatment, even if it cannot delay the disease progression, has a symptomatic effect on some cognitive, psychological, and behavioral symptoms. 

The targets of drugs with regulatory indication for symptomatic treatment of AD are the cholinergic system and the glutamatergic systems. Acetylcholinesterase/cholinesterase (AChE/ChE) inhibitors (Is) increase acetylcholine levels by reducing the breakdown of the neurotransmitter, whereas memantine antagonizes N-methyl-D-aspartate (NMDA) receptors [2]. Memantine is a moderate affinity, uncompetitive antagonist of NDMA receptors. It alleviates to some extent the behavioral symptoms of Alzheimer's disease, with benefits on cognitive, functional, and global status [3]. Memantine activity is explained by the diffusion of NMDA receptors which are more abundant in the hippocampus and in the cerebral cortex, the brain areas more largely involved in cognition, learning, and memory. Glutamate or glutamic acid mediates long-term potentiation via NMDA receptors. Elevated glutamate levels are associated with the development of neurotoxicity phenomena and this could explain the beneficial effect of memantine in the blocking of the negative consequences of elevated glutamate levels. After initial skepticism, both the National Institute for Clinical Excellence (NICE) and the IQWIG (the German Institute for Quality and Efficiency in Healthcare) revised their original conclusions and recommended memantine in AD, primarily of the moderate-to-severe stage [4, 5]. The recommended starting dose is 5 mg daily, with 5 mg increments weekly, up to a maximum of 10 mg twice a day. Memantine is well tolerated; adverse effects are uncommon and no more frequent than placebo. They include dizziness, confusion, somnolence, hallucinations, and nausea which disappear after discontinuation or dose reduction [6, 7]. 

AChEIs/ChEIs are considered the standard treatment of the mild-moderate stage of AD [8]. They act enhancing the cholinergic transmission through the inhibition of AChE/ChE, the enzymes degrading acetylcholine in the synaptic cleft to choline and acetate. Slowing down of the acetylcholine catabolism makes neurotransmitters more available. Three AChEIs/ChEIs are on the market: donepezil, rivastigmine, and galantamine. All have demonstrated a small but measurable clinical benefit [9, 10]. Donepezil is approved in the mild-to-moderate AD stage in Europe and Japan and in all stages of the disease in the United States (USA) and some other countries. In 2010, the US Food and Drug Administration (FDA) has also approved the use of the compound at the daily dose of 23 mg/day for treating patients in the moderate-to-severe stage of AD [11]. It has been reported that this dose provides a small but significant improvement in the cognitive endpoints compared with the 10 mg/day dose [12, 13].

The use of an association of donepezil with memantine for moderate-severe AD is more recent [14]. Some studies reported that it may be effective to slow down the cognitive and functional decline, reducing therefore the nursing home admissions beyond what is found with single drugs [15]. 

The aim of this paper was to review clinical trials using memantine, donepezil, or the two drugs in association in managing moderate-severe AD. In particular we wanted to answer to the following questions.

- What is the documented benefit using memantine or donepezil in moderate-severe AD subjects?
- Are these drugs safe and manageable in these subjects?
- Which doses should be used?
- Is there any advantage in using the two drugs in association?

Analysis included and compared double-blind, placebo-controlled, randomized controlled trials (RCTs) evaluating memantine in monotherapy, donepezil in monotherapy, and the association of memantine plus donepezil in managing the symptoms of patients with moderate-severe AD. Our first aim was to clarify if the association of two drugs might be more beneficial than single treatment. The second aim was to identify drugs and doses more indicated in monotherapy in moderate-severe Alzheimer's patients.

## 2. Methods

Reports of double-blind, placebo-controlled RCTs using memantine or donepezil alone, or in association versus placebo were identified using PubMed and Medline databases. Analysis was done in January 2013 and included the 5-year period between 2007 and 2012. Entries used were (1) memantine for moderate-to-severe AD; (2) donepezil for moderate-to-severe AD; (3) donepezil and memantine for moderate-to-severe AD; (4) memantine in patients with severe AD; (5) donepezil in patients with severe AD; (6) donepezil and memantine in patients with severe AD. 

The title and abstract of each article were first examined and only “good quality” papers were included. For fulfilling “good quality” criteria, papers should be in English and should have an abstract and at least one of the key words above indicated in the title/abstract. Only RCTs and meta-analyses were considered. 

The list of criteria for the eligible studies is reported in Table 1. 

Articles were further selected using the Newcastle-Ottawa Scale criteria [16]. This scale allows assessing the methodological quality of comparative and case control studies based on published materials. Clinical and demographic characteristics and the outcomes referring to moderate and severe AD patients from the trial reports were extracted. 

Other outcomes of interest were the clinical global impression, the cognitive function, the functional performance in daily life activity (ADL), and the behavioral symptoms. Instruments for the cognitive assessment included: the Mini-Mental State Examination (MMSE), the Clinician's Interview-Based Impression of Change plus caregiver's input (CIBIC-plus), the AD Assessment Scale-Cognitive subscale (ADAS-Cog), Severe Impairment Battery (SIB), the AD Cooperative Study-Activities of Daily Living (ADCS-ADL) scale (19- and 23-item), and the Neuropsychiatric Inventory (NPI) (Table 2). 

To compare the results obtained by different studies a regression algorithm evaluating the progression of the degenerative disease and the influence of treatments on it was also used. 

## 3. Results

Only 83 of the 941 abstracts selected were considered relevant to the topic, and only 13 met the criterion of “adequacy and representativeness” indicated by Newcastle-Ottawa score. Therefore, the results reported here refer to data emerging from these 13 studies. They include 6 RCTs with memantine as monotherapy, 5 RCTs with donepezil as monotherapy, and 2 RCTs with donepezil plus memantine treatment. 

Comparative analysis of data of different trials with the regression algorithm of results obtained was not possible due to the short time of the studies that except one reaching one year of observation did not exceed 6 months.

### 3.1. Memantine Monotherapy

The 6 trials that examined the effect of memantine in moderate-to-severe AD patients are listed in Table 3. 

The baseline characteristics of patients recruited by these 6 RCTs are summarized as follows.

Fox et al. [17]: 149 participants recruited from nursing or residential care homes and acute psychiatric wards in the United Kingdom, mean age 85 years (memantine groups) and 84 years (placebo groups) and mean baseline MMSE 7 in both groups. 

Förstl et al. [18]: 4305 participants recruited from different sites in Greece and Germany, mean age 75 years and mean baseline MMSE 17. 

Herrmann et al. [19]: 24 participants were recruited from two LTC facilities (a Veteran Affairs Canada LTC facility and a community nursing home), assessed for 3 months, mean age 86 years and mean baseline MMSE 9. 

Schulz et al. [20]: 93 patients were screened and treated at 21 study centers in Germany and Austria, assessed for 12 weeks, mean age years 74 and mean baseline MMSE 16.

Rainer et al. [21]: 350 participants recruited from 88 Austrian centers (general practitioners, psychiatric and neurological practices, and outpatient hospital departments), assessed for 4 months, mean age years 78 and mean baseline MMSE 12.

Wilcock et al. [22]: 593 participants, assessed 6 months, mean age years 77 and mean baseline MMSE 9.

The general conclusion of different investigations was that treatment with memantine improved global cognition [18, 20, 21] and functional communication [20] and had positive effect on agitation and aggressive behavior [19–22]. The effect on agitation was not confirmed by one study [17]. A pooled analysis of moderately severe to severe AD subjects (MMSE 3 to 14) showed that in subjects having at baseline symptoms of agitation/aggression or psychosis memantine induced an improvement over 6 months. This effect was significantly greater than that found in patients treated with placebo [22].

### 3.2. Donepezil Monotherapy

Trials with donepezil on moderate-to-severe AD patients are summarized in Table 4. The baseline characteristics of these studies are summarized as follows.

Carrasco et al. [23]: 455 participants recruited from 72 centers from Spain, assessed for 24 weeks, mean age 74 years, mean baseline MMSE 16 (moderate) and 11 (severe). Concomitant medications excluded were anticholinergic drugs, cholinergic agonists, neuromuscular blocking agents, and beta blockers.

Farlow et al. [24, 25]: 1,434 participants recruited from 219 sites in Asia, Europe, Australia, North America, South Africa, and South America, assessed for 24 weeks, mean age 74 years, mean baseline MMSE 13. 

Schwam and Xu [26]: 290 participants recruited from 37 US sites, in the community or assisted-living facilities, mean age 73.3 (donepezil group) and 74 years (placebo group), mean baseline MMSE 11.72 (donepezil group) and 11.97 (placebo group). 

Homma et al. [27]: 325 participants recruited from Tokyo, Japan, assessed (primary) for 24 weeks, mean age 78 (donepezil 5 mg group), 77 (donepezil 10 mg group), and 80 years (placebo group), mean baseline MMSE 8 (donepezil 5 mg group), 7 (donepezil 10 mg group), and 8 (placebo group). 

Black et al. [28]: 343 participants recruited from 98 centers in the United States, Canada, France, the United Kingdom, and Australia, mean age 78 years, mean baseline MMSE 7. 

The first unambiguous demonstration of donepezil's beneficial impact on behavioral symptoms in patients with the moderate-severe stage was provided by Carrasco and coworkers [23]. The main effects included the improvement of neuropsychiatric and cognitive functions and the reduction of caregiver burden. In a post hoc exploratory analysis, the largest decline in the ability to perform basic activities of daily living (BADLs) occurred in the placebo group of patients with a baseline SIB score of 70. Changes were reduced in the donepezil-treated group. This study suggests that benefits in functional abilities are associated with a good level of cognition [26]. In patients with severe AD, donepezil had a greater efficacy than placebo on cognition and global function measures [27, 28]. Among all the trials analyzed only two studies have assessed in detail the safety and tolerability of donepezil at 23 mg/day compared with 10 mg/day. These investigations demonstrated safety and the predictable tolerability profile for the higher dose and therefore support the use of high doses of donepezil in moderate-to-severe AD [24, 25]. 

### 3.3. Donepezil Plus Memantine Treatment

Trials examining and assessing the effect of treatment with donepezil plus memantine on moderate-to-severe AD patients are summarized in Table 5. The baseline characteristics of the patients recruited by the 2 RCTs using donepezil + memantine for moderate-severe AD are summarized as follows.

Howard et al. [29]: 295 community-dwelling patients, assessed for 52 weeks, mean age 77 and mean baseline MMSE 9 in both groups. 

Doody et al. [30]: 1434 participants, of which 520 patients treated with donepezil and memantine, recruited from 219 sites in Asia, Europe, Australia, North America, South Africa, and South America, assessed for 24 weeks, mean age 74 years and mean baseline MMSE 14.

These two RCTs have investigated the efficacy and safety of memantine 20 mg/day in combination with a ChEI in moderate-to-severe AD patients (Table 5). In both studies, the combination therapy was well tolerated. The first study [29], has shown that treatment with memantine and donepezil was associated with cognitive benefits exceeding the minimal clinically important difference and that significant functional benefits were found in the 12 subsequent months. Combined treatment (donepezil and memantine) was not significantly superior to treatment with donepezil alone with respect to any of the primary or secondary outcomes. Hence this investigation did not show significant benefits of the combination of donepezil and memantine over donepezil alone. 

The second RCT [30] has assessed the efficacy of donepezil 23 mg versus donepezil 10 mg, for moderate-to-severe Alzheimer's, but patients were stratified by the concomitant memantine use. In this population, the concomitant memantine use did not alter the response profile of donepezil 23 versus 10 mg/day. 

## 4. Discussion

According to DSM-IV and NINCDS-ADRDA criteria, an AD course has to be distinguished in mild, moderate, and severe stages. The first refers to subjects having MMSE score between 21 and 26, the second refers to those with MMSE ranging from 20 to 10, and the third (severe stage) refers to subjects with MMSE score under 10. Worthy to note is that the duration of each stage is different too. The mild stage has a mean duration of 2–4 years, the moderate stage has a much longer duration ranging from 2 to 10 years, and the severe stage duration is of about 3 yrs. On referring to the moderate-severe stage we are therefore taking into account the longest part of the disease course. As already mentioned in the introduction, treatments approved for AD include to the largest extent AChE/ChEIs (donepezil, rivastigmine, and galantamine) and the NMDA receptors antagonist memantine [31]. All treatments have symptomatic effects lasting for a long period, but none has been demonstrated to delay the global disease duration [32, 33]. 

The moderate-severe AD subjects show that cognitive and functional abilities and social interactions are severely reduced, their capacity to perform instrumental activities of daily life (IADLs) is impaired, and only some ADLs may be still carried out. Bathing and toileting usually need aid [34] and obviously these subjects are those requiring more care and resources. For treating AD at this stage the only approved treatments are memantine (worldwide) and donepezil (in the USA). Donepezil has less evident advantages in the severe stage than in the mild-to-moderate stage. The first prospective study done in patients with the severe stage and demonstrating that donepezil is beneficial on cognitive and global functions is a 6-month observation conducted in nursing homes in Sweden and has been recently published [35]. Other studies have reported that the clinical worsening (of cognition, global functions, and global status) is less pronounced in patients treated with donepezil. Similar results are found with memantine. Patients treated with it in monotherapy showed less worsening than the placebo group [36, 37]. On assessing the results of monotherapy treatment of moderate-severe AD patients, RCTs reviewed indicate that both memantine and donepezil show benefits. These benefits are in cognitive [18, 20, 21, 27, 28] and global outcomes [20, 26] and in behavioral symptoms [19, 22, 23].

The benefit of combining memantine with ChEI therapy in the moderate stage of AD is less evident. Some studies conducted in clinical settings show that the association led to improvements [14, 38–40] or delayed worsening [18]. It has been also reported that benefits increased with treatment persistence and that they extended across multiple domains [33]. Analysis of RCTs we have reviewed does not confirm such effect and the DOMINO-AD study [29] does not show significant benefits of the combination of donepezil and memantine over donepezil alone. This is true both memantine was associated with donepezil 10 mg [29] or 23 mg [30].

Some methodological limitations of the DOMINO-AD study, which has been taken into account in our review, may explain the different conclusions between our results and other studies. The DOMINO-AD study [29] required a redesign because of the delayed and insufficient recruitment. Hence, these results may have been substantially biased towards null results, failing to demonstrate significant differences between donepezil only and the association of donepezil plus memantine. Our conclusions are similar to those reached by NICE in 2010 [41], of no additional benefit of the combination memantine plus AChE/ChE-Is versus memantine monotherapy, and to those of another study covering 1 year [42]. Opposite conclusions for the cognition domains, everyday functions, and global status were reported by a post hoc metanaalysis [43, 44]. Unfortunately, due to the heterogeneity of conditions explored by RCTs and of the cognitive assessment tools used, it is not possible to compare properly the different trials. This is the reason why our work was limited to a descriptive analysis. As a general consideration it should be pointed out that the time of patient's observation was limited to 24 and 52 weeks. Considering the time course of moderate stages of AD, a longer time of observation would be desirable to provide a reliable measure of the advantages of any treatment. 

During our work we have tried to conduct a pharmacoeconomic evaluation between the association, memantine plus donepezil and memantine or donepezil alone. This analysis did not lead to satisfactory results because studies available considered different statistical models and samples, nonhomogeneous criteria for inclusion and exclusion of patients, and heterogeneous clinical endpoints. All these differences did not allow the comparison and evaluation of the economic impact of treatments under analysis. On the other hand, clinical studies analyzed in this review apparently excluded associated diseases probably affecting patients, which are conditions known to influence the weight and impact of the total costs of the disease [45]. 

In conclusions, the evaluation of RCT results suggests that both memantine and donepezil lead to improvements in moderate-to-severe AD. Memantine was found to improve global cognition [18, 20, 21], functional communication [20], and some behavioral symptoms (agitation and aggression) [19, 22]. Donepezil at 10 mg/day improved the neuropsychiatric [23], cognitive [23, 26–28], and global functions [26–28], reducing therefore the caregiver burden [23]. Donepezil at 23 mg/day was found to be safe and tolerated in patients with moderate-to-severe AD [24, 25], but further studies are necessary for confirming its suitability in the severe stage of the disease. Both drugs are therefore indicated for the treatment of moderate AD and the choice between them should be based on their contraindications more than on disease severity. Inversely, on the basis of the RCTs we reviewed, no evidence is found for the advantages of the association of memantine with AChE/ChE-Is. 

## Figures and Tables

**Table 1:** Inclusion and exclusion criteria of papers selected for this review.

Criterion	Reason of inclusion	Reason of exclusion
Population	Qualifying disease: AD (diagnosed with established criteria, e.g., DSM-IV and NINCDS-ADRDA) Any severity of disease at baseline Community-/nursing-home-dwelling residents	

Perspective of the study	Prospective (concurrent) Comparative	Retrospective (nonconcurrent, historical)

Type of the study	RCT (open label or blinded) Crossover trials with a washout period between treatments	Non-randomised CCT: cohort observational case control cross sectional noncomparative study

Language	English	All others

Study duration	Any	None

Sample size	Any	None

Intervention/treatments	Any dose of (i) donepezil (ii) memantine	

Control intervention/treatments	Placebo/usual care Any of the above interventions	

DSM-IV: Diagnostic and Statistical Manual of Mental Disorders; NINCDS-ADRDA: National Institute of Neurological and Communicative Disorders and Stroke and the AD and Related Disorders Association; CCT: controlled clinical trial.

**Table 2:** Assessment scales mainly used in the clinical trials reviewed.

Scale acronym	Definition	Description
ADAS-Cog	Alzheimer's Disease Assessment Scale Cognitive Subscale	Standard instrument for measuring cognitive ability on an 11-item scale in patients with mild-to-moderate disease. Scores range from 0 to 70, with higher scores indicating higher impairment

ADCS-ADL	Alzheimer's Disease Cooperative Study- Activities for Daily Living	Assessment of functional abilities on a 19-item, 54-point scale modified for moderate-to-severe dementia (a 23-item scale is used for mild-to-moderate disease subjects). A higher score indicates better functioning

CIBIC-plus	Clinician's Interview-Based Impression of Change plus	Caregiver input evaluates overall global change relative to the baseline level in cognitive, functional, and behavioural aspects. The scale ranges from 1 (very much improved) to 7 (very much worsened)

FAST	Functional Assessment Staging	Evaluates dementia progression from stage 1 (normal) to 7 (severe dementia)

GDS	Global Deterioration Scale	Evaluates overall cognitive and functional capacity on a 7-stage scale, based on the patient and caregiver assessment. Higher stages indicate greater impairment

MMSE	Mini-Mental State Examination	Evaluates the cognitive function on a 30-point scale. A higher score indicates a better function

NPI	Neuropsychiatric Inventory	Caregiver-rated assessment that evaluates the patient's behaviour on a 12-item, 144-point scale. A lower score indicates a better behavior

SIB	Severe Impairment Battery	Evaluates the cognitive dysfunction on a 40-item, 100-point scale in patients with moderate-to-severe Alzheimer's disease. A higher score indicates better cognitive functioning

**Table 3:** Clinical trials examined assessing the influence of treatment with memantine in moderate-severe AD.

First author and no. in the list of references	Severity	Mean age years	Mean MMSE at baseline	Duration of the study	*N*	Test
Fox et al. 2012 [17]	MMSE ≤ 19	Placebo: 84.4 (6.6) Memantine: 84.9 (6.7)	Placebo: 7.3 (6.4) Memantine: 7.3 (6.2)	12 weeks	149	MMSE SIB NPI CMAI CGI-C

Förstl et al. 2011 [18]	MMSE ≤ 27	75.5 ± 7.5	17.1 ± 5.5	6 months	4305	MMSE IADL

Herrmann et al. 2011 [19]	MMSE 0–15	85.81 ± 3.7	8.74 ± 6.71	3 months	24	NPI CGI-C

Schulz et al. 2011 [20]	MMSE ≤ 20	Memantine: 77.8 ± 8 Placebo: 76 ± 6	16.0 (3.7)	12 weeks	93	CERAD-NP ACDS-ADL-sev CGI-C

Rainer et al. 2011 [21]	MMSE < 20	77.6 ± 7.569	11.75 ± 6.38	4 months	350	MMSE CGI

Wilcock et al. 2008 [22]	MMSE 14–3	Memantine: 76.9 (8.4) Placebo: 76.7 (7.9)	Memantine: 9.2 (3.3) Placebo: 9.6 (3.1)	6 months	593	MMSE SIB NPI ACDS-ADL-sev CIBIC-plus

**Table 4:** Clinical trials on treatment with donepezil in moderate-severe AD.

First author and number in the list of references	Severity	Mean age years	Mean MMSE at baseline	Duration of the study	*N*	Test
Carrasco et al. 2011 [23]	MMSE 1–23	74.4 (7.7)	Mild: 20.14 (4) Moderate: 15.9 (3.83) Severe: 11.3 (5.4)	6 months	455	MMSE NPI

Farlow et al. 2011, 2010 [24, 25]	MMSE 0–20	10 mg: 73.8 (8.56) 23 mg: 73.9 (8.53)	10 mg: 13.0 (4.75) 23 mg: 13.1 (4.99)	24 weeks	1434	MMSE ACDS-ADL-sev SIB CIBIC-plus

Schwam and Xu 2010 [26]	MMSE 5–17	Placebo: 74 Donepezil: 73.3	Placebo: 11.97 ± 0.34 Donepezil: 11.72 ± 0.35	24 weeks	290	NPI SIB CIBIC-plus MMSE DAD

Homma et al. 2008 [27]	MMSE |1–12|	Placebo: 79.7 ± 7.5 5 mg: 78.0 ± 8.9 10 mg: 76.9 ± 7.9	Placebo: 8.0 ± 3.3 5 mg: 7.9 ± 3.3 10 mg: 7.4 ± 3.4	24 weeks	325	BEHAVE-AD ACDS-ADL-sev CIBIC-plus MMSE SIB

Black et al. 2007 [28]	MMSE |1–12|	78.0 (8.10)	Donepezil: 7.5 (325) Placebo: 7.4 (3.57)	24 weeks	343	ACDS-ADL-sev CIBIC-plus MMSE SIB NPI CBQ

**Table 5:** Clinical trials characteristics and results of the association of donepezil plus memantine in moderate-severe AD.

First author and no. in the list of references	Severity	Mean age years	Mean MMSE at baseline	Duration of the study	*N*	Test
Howard et al. 2012 [29]	MMSE 5–13	77.1 ± 8	9.1 ± 2.6	52 weeks	295	MMSE BADLS NPI PROXY

Doody et al. 2012 [30]	MMSE 0–20	Memantine: 73.6 (8.63) Placebo: 74.0 (8.48)	13.8 (4.63)	24 weeks	1436	MMSE CIBIC-plus ACDS-ADL-sev SIB
